# Growth-Temperature-Dependent Synaptic Plasticity in ALD-ZnO Optoelectronic Synaptic Devices on Polyimide Substrates

**DOI:** 10.3390/s26196022

**Published:** 2026-09-23

**Authors:** JunHyeong Park, JungBeen Cho, SeungMin Song, Seyeon Tak, Yoon Seok Kim, Sung-Nam Lee

**Affiliations:** 1 Department of Nano and Semiconductor Engineering, Tech University of Korea, Siheung 15073, Republic of Korea; 2 Department of Semiconductor Engineering, Tech University of Korea, Siheung 15073, Republic of Korea

**Keywords:** ALD, synapse, ZnO, polyimide, growth temperature

## Abstract

**Highlights:**

**What are the main findings?**

- Higher ZnO growth temperatures enhanced photocurrent, PPC, and synaptic memory.
- Surface morphology and defect-related properties strongly influenced carrier retention.

**What are the implications of the main findings?**

- ALD growth temperature enables simple control of synaptic plasticity on flexible PI.
- Flexible ZnO devices are promising for wearable neuromorphic and visual-memory applications.

**Abstract:**

Flexible Al/ZnO/Al optoelectronic synaptic devices were fabricated on flexible polyimide (PI) substrates using ZnO thin films deposited by atomic layer deposition (ALD) at growth temperatures ranging from 90 to 145 °C. The effects of growth temperature on the surface morphology, optical properties, electrical transport, and synaptic behavior were systematically investigated. All ZnO films were grown at temperatures compatible with polymer substrates, while their surface roughness, photoluminescence, optical transmittance, dark current, and photocurrent varied considerably with growth temperature. Increasing the growth temperature enhanced carrier transport, photocurrent, and persistent photoconductivity, resulting in larger excitatory postsynaptic currents (EPSCs) and slower post-illumination decay. The EPSC was further modulated by the ultraviolet (UV) stimulation duration, light intensity, and pulse number. Paired-pulse facilitation decreased with increasing pulse interval, whereas higher-temperature-grown devices maintained stronger facilitation over longer intervals. Repeated optical stimulation enabled faster relearning and slower forgetting, thereby mimicking learning-experience behavior. Moreover, 5 × 5 visual-memory tests showed growth-temperature-dependent image retention, with the 145 °C device exhibiting the slowest pattern decay. These results demonstrate that ALD growth-temperature engineering provides an effective strategy for tuning synaptic plasticity and visual-memory retention in flexible ZnO-based optoelectronic devices.

## 1. Introduction

With the rapid development of artificial intelligence, neuromorphic computing has attracted considerable attention because of its potential for processing large amounts of information with low energy consumption [1,2,3]. Conventional von Neumann computing systems physically separate the processor and memory, requiring continuous data transfer between the two units. This architecture causes latency and energy consumption, particularly during data-intensive operation [1,4,5]. Artificial synaptic devices have therefore been developed to emulate the signal transmission, learning, and memory functions of biological synapses while integrating information processing and storage within a single device [6,7]. Among various artificial synaptic systems, optoelectronic synaptic devices use light as a presynaptic stimulus and convert optical information directly into an electrical postsynaptic response [2,3]. Optical inputs provide several advantages, including high bandwidth, low crosstalk, and the ability to regulate the synaptic response through wavelength, duration, interval, and repetition number of the light pulses [2,3]. The resulting photoinduced current can emulate excitatory postsynaptic current (EPSC), paired-pulse facilitation (PPF), short-term memory, and long-term memory [6,7,8]. These device characteristics correspond to temporal synaptic plasticity, whereas the biological basis of short- and long-term memory has been described separately in neuroscience studies [8].

ZnO is a promising active material for optoelectronic synaptic devices because of its wide bandgap, high transparency in the visible region, and strong ultraviolet photoresponse [9,10]. Several ZnO-based devices have demonstrated optically induced synaptic plasticity and persistent current retention under ultraviolet (UV) stimulation [11,12,13]. When ZnO absorbs UV light, electron–hole pairs are generated, increasing the carrier concentration and electrical current. However, the photoresponse is also strongly influenced by surface adsorption processes and defect-mediated carrier trapping. Under dark conditions, adsorbed oxygen molecules capture free electrons from ZnO, whereas UV-generated holes promote oxygen desorption and release electrons back into the conduction process. In addition, oxygen vacancies and other defect states can trap photogenerated carriers and delay their recombination [12,14]. These processes produce persistent photoconductivity (PPC), in which the current remains above its initial value after the optical stimulus is removed. PPC is therefore directly related to the decay dynamics, carrier-retention time, and memory characteristics of ZnO optoelectronic synaptic devices [11,12,13]. In addition to thin-film deposition techniques, ZnO nanostructures have also been prepared by solution-based methods, including co-precipitation and hydrothermal growth. ZnO nanoparticles and hierarchical nanorod structures synthesized by these approaches have exhibited pronounced photoconductive responses, with their photocurrent characteristics strongly influenced by morphology, defect states, and surface-related processes [15,16]. The morphology, defect distribution, and carrier-transport properties of ZnO strongly depend on the preparation condition. Thermal processing has been shown to alter the microstructural, optical and electrical properties of ZnO nanomaterials and to modify their synaptic memory characteristics [17]. Nevertheless, these previous studies mainly examined post-deposition thermal treatment, nanoparticle-based devices, or ZnO structures fabricated on rigid substrates. The effect of the direct atomic layer deposition (ALD) growth temperature on the photoresponse and short- and long-term memory characteristics of ZnO films deposited on flexible polymer substrates remains insufficiently understood. In particular, the relationship between low-temperature ALD growth conditions, persistent photoconductivity, and the resulting synaptic plasticity in flexible ZnO devices has not yet been systematically clarified. Accordingly, determining how the ZnO growth temperature controls carrier transport, defect-related relaxation, and PPC is important for optimizing flexible optoelectronic synaptic devices. Flexible synaptic devices are particularly attractive for wearable electronics, electronic skin, and conformable sensing systems [18,19]. Polyimide (PI) is widely used as a flexible substrate because of its mechanical durability and thermal stability. However, its dimensional stability and surface properties can deteriorate during excessive thermal processing, requiring the active layer to be deposited within a relatively low-temperature range. ALD is suitable for this purpose because it provides conformal and thickness-controlled films through sequential, self-limiting surface reactions [20,21,22,23]. ALD-grown ZnO can also exhibit growth-temperature-dependent electrical properties, reflecting variations in precursor reaction completeness, impurity incorporation, bonding configuration, and defect formation [24]. At excessively low growth temperatures, incomplete surface reactions may increase residual ligands and structural disorder, whereas higher temperatures may promote more complete reactions and improved carrier-transport pathways. Although the UV-induced photoresponse and PPC mechanism of ZnO are well established, the novelty of this work lies in demonstrating a simple process-level strategy for tuning synaptic plasticity and memory dynamics solely through the low-temperature ALD growth temperature on a flexible PI substrate. In particular, we systematically investigate how direct ALD growth within a PI-compatible temperature range of 90–145 °C, without post-deposition annealing, modulates carrier relaxation and PPC and, consequently, EPSC, PPF, learning–forgetting behavior, and visual-memory retention. This study demonstrates that controlling the low-temperature ALD growth conditions of ZnO is an effective strategy for tuning carrier transport, persistent photoconductivity, synaptic plasticity, and visual-memory retention in flexible Al/ZnO/Al optoelectronic synaptic devices.

## 2. Materials and Methods

Flexible ZnO optoelectronic synaptic devices were fabricated on commercial PI substrates. Prior to ZnO deposition, the PI substrates were sequentially cleaned with acetone and isopropyl alcohol, rinsed with deionized water, and dried with N_2_ gas. ZnO thin films were deposited by ALD (ATOMIC CLASSIC, CN1, Gyeonggi-do, Republic of Korea) at growth temperatures of 90, 110, 130, and 145 °C. The ALD growth rate had been calibrated in preliminary process-optimization experiments using X-ray reflectivity (XRR), and 500 ALD cycles were subsequently employed for all growth temperatures to obtain ZnO films with a comparable final thickness of approximately 50 nm. This minimized possible thickness-related contributions to the growth-temperature-dependent electrical and optoelectronic characteristics. Diethylzinc (DEZn) and H_2_O were used as the Zn and oxygen precursors, respectively, while N_2_ was employed as the purge gas. Each ALD cycle consisted of four sequential steps: DEZn pulse, N_2_ purge, H_2_O pulse, and N_2_ purge, with each step maintained for 0.25 s. During deposition, the N_2_ flow rate was maintained at 1600 sccm, and the process pressure was approximately 1.1 × 10^−1^ Torr. The combination of the high N_2_ flow rate and low process pressure facilitated rapid precursor evacuation and gas exchange between the sequential DEZn and H_2_O exposures. After ZnO deposition, circular Al electrodes were formed on the ZnO surface by thermal evaporation through a shadow mask. The Al electrodes had a thickness of approximately 50 nm and a diameter of 200 μm, with a lateral spacing of 200 μm between adjacent electrodes. The effective UV-illuminated area between the electrodes was approximately 4.86 × 10^−4^ cm^2^. The resulting planar Al/ZnO/Al structure allowed electrical current to flow laterally through the ZnO layer between adjacent Al electrodes. All devices were fabricated under identical deposition and electrode-formation conditions, except for the ZnO growth temperature, allowing the influence of growth temperature on the electrical, optoelectronic, and synaptic characteristics to be systematically evaluated.

The morphological, optical, electrical, and optoelectronic properties of the ZnO films and devices were systematically characterized. The surface morphology of the ZnO films was examined using atomic force microscopy (AFM; NanoFocus my-Scope plus, Seoul, Republic of Korea). Three-dimensional surface images and root-mean-square (RMS) roughness values were obtained to evaluate the influence of ALD growth temperature on the ZnO surface structure. The RMS roughness was evaluated at five different regions of each sample, and the values are presented as the mean ± standard deviation. The optical properties of the ZnO/PI samples were investigated using a UV–visible spectrophotometer (Evolution 300, Thermo Fisher Scientific, Waltham, MA, USA). Room-temperature photoluminescence (PL) spectra were measured under 266 nm laser excitation to investigate defect-related visible emission from the ZnO films. The electrical and optoelectronic characteristics of the fabricated devices were measured using a Keithley 2614B (Keithley Instruments, Cleveland, OH, USA) source-measure unit connected to a probe station. Current–voltage characteristics were recorded under dark conditions and under 365 nm UV illumination. The photocurrent was determined from the difference between the UV-illuminated current and the dark current. In addition, time-dependent current responses were recorded during and after UV illumination to evaluate the PPC behavior. For each growth condition, the electrical, optoelectronic, and synaptic characteristics were evaluated using 10 independently fabricated devices. The reported statistical values are presented as the mean ± standard deviation, with corresponding error bars included in the relevant figures. These measurements were used to correlate growth-temperature-dependent changes in surface morphology and optical properties with the electrical transport and photoresponse characteristics of the ZnO devices.

## 3. Results and Discussion

### 3.1. Effects of ALD Growth Temperature on the Morphological and Optical Characteristics of ZnO Films on PI Substrates

Figure 1a illustrates the structure of the ZnO thin film deposited by ALD on a flexible PI substrate. The ZnO films were grown at temperatures ranging from 90 to 145 °C to investigate the effects of growth temperature on their surface morphology and optical properties. The AFM images shown in Figure 1b reveal that all ZnO films exhibited bump-like surface features, although their size, distribution, and height varied considerably with growth temperature. The films grown at 90 and 110 °C exhibited relatively small and densely distributed surface features, whereas larger and less uniformly distributed structures developed at the higher growth temperature. This morphological evolution was most noticeable in the film grown at 145 °C, where larger agglomerated surface features became more prominent. The RMS roughness values were obtained from five different regions of each sample and are presented as the mean ± standard deviation (Figure 1c). The AFM images also show a gradual increase in the lateral size and height variation of the surface features with increasing growth temperature. However, because AFM alone cannot conclusively determine the microscopic growth mechanism responsible for this morphological evolution, the observed increase in roughness is described here only as a growth-temperature-dependent change in the surface morphology rather than being directly attributed to enhanced surface diffusion, precursor reaction completeness, or coalescence of neighboring nuclei [25]. Figure 1d shows the ω–2θ XRD patterns of the ZnO films grown on PI at different temperatures. A diffraction peak corresponding to the ZnO (002) plane is observed at approximately 34.4° for all growth temperatures, confirming the formation of the hexagonal wurtzite ZnO phase [10]. Because the ZnO films had comparable thicknesses of approximately 50 nm for all growth conditions, the variation in the (002) peak intensity is not primarily attributable to film-thickness differences. Instead, the progressively more pronounced (002) reflection with increasing growth temperature indicates an enhanced preferential orientation of the ZnO crystallites along the c-axis and is consistent with improved structural ordering at higher growth temperatures. A very weak diffraction feature near 36.2°, corresponding to the ZnO (101) plane, becomes discernible for the film grown at 145 °C, whereas it is not clearly resolved for the films grown at lower temperatures. This suggests that the ZnO films are predominantly oriented along the (002) direction over the investigated temperature range, while a minor contribution from other crystallographic orientations may emerge at the highest growth temperature. As shown in Figure 1e, all ZnO/PI samples exhibited a broad visible PL emission band extending from approximately 500 to 700 nm at room temperature. This broad emission is more generally associated with defect-related radiative recombination involving a distribution of sub-bandgap states, rather than being uniquely assigned to specific defect species [26]. Because these emission components strongly overlap, room-temperature PL alone does not allow an unambiguous identification or quantitative separation of individual defect-related transitions. The PL intensity increased with decreasing growth temperature, with the film grown at 90 °C showing the strongest deep-level emission. This variation indicates a growth-temperature-dependent change in the overall defect-related radiative recombination; however, the PL intensity should not be interpreted as a direct measure of the concentration of any specific defect species. At lower growth temperatures, incomplete surface reactions and less-developed Zn-O bonding may contribute to a larger population of radiatively active sub-bandgap states, whereas increasing growth temperature may modify the local bonding environment and reduce the overall defect-related visible emission [24,25,26]. Figure 1f presents the UV–visible transmittance spectra of the ZnO/PI samples. Transmittance was evaluated because it directly represents the fraction of incident light passing through the combined ZnO/PI structure. All samples showed low transmittance below approximately 480 nm, followed by a rapid increase near 500 nm, reaching approximately 60–75% at 800 nm. The films grown at lower temperatures generally exhibited slightly higher transmittance. However, the overall spectral response was influenced by both the ZnO layer and the optically absorbing PI substrate. Because the colored PI substrate strongly absorbs ultraviolet and short-wavelength visible light, the transmittance remained low below approximately 500 nm and increased at longer wavelengths as the substrate became more transparent [27]. 

Accordingly, the measured spectra should be interpreted as the optical response of the combined ZnO/PI structure rather than as the intrinsic optical response of the ZnO layer alone. In particular, the strong absorption of the PI substrate in the UV and short-wavelength visible region obscures the intrinsic ZnO absorption edge, making a reliable determination of the ZnO optical bandgap from these spectra difficult. Above 550 nm, the small transmittance differences among the samples may be associated with the changes in surface morphology, scattering, and defect-related optical absorption; however, these contributions cannot be quantitatively separated from the present transmittance measurements alone. The stronger PL of the low-temperature-grown films indicates enhanced radiative defect recombination, whereas the lower transmittance at higher temperatures is consistent with the increased surface roughness and larger surface features observed by AFM. Overall, increasing the ZnO growth temperature produced a clear evolution of the surface morphology and a substantial increase in surface roughness, accompanied by changes in the visible PL intensity and optical transmittance of the ZnO/PI structures.

### 3.2. Growth-Temperature-Dependent Electrical and Photoresponse Characteristics of Flexible Al/ZnO/Al Devices

Figure 2a shows the structure of the planar Al/ZnO/Al optoelectronic synaptic device fabricated on a flexible PI substrate, where circular Al electrodes were formed on the ZnO film grown by ALD. In this lateral device configuration, the current flows through the ZnO channel between two adjacent Al electrodes. The electrical properties were measured in the dark and under 365 nm UV illumination to investigate the influence of the ZnO growth temperature on charge transport and photoresponse behavior. As shown in Figure 2b, the devices fabricated using ZnO films grown at different temperatures exhibited nearly symmetric current–voltage (I–V) characteristics in positive and negative voltage regions, primarily because both contacts consisted of the same Al electrode material. The dark current clearly increased with growth temperature, showing the lowest value in the 90 °C device and the highest value in the 145 °C device. At lower growth temperatures, structural disorder and discontinuous or poorly connected transport pathways may limit electrical conduction. In contrast, higher temperature can promote more complete ALD surface reactions and atomic rearrangement, leading to improved Zn–O bonding and more continuous carrier-transport pathways [24,25]. Figure 2c presents the I–V characteristics measured under 365 nm UV illumination, with higher currents observed for devices grown at higher temperatures. When ZnO absorbs 365 nm light, electron–hole pairs are generated, and the photogenerated electrons contribute to electrical conduction, thereby increasing the current [12,13,14]. In the ZnO films grown at higher temperatures, these carriers may be transported and collected more efficiently owing to improved film connectivity and electrical transport pathways [24]. Figure 2d shows the photocurrent (I_Photo_) calculated from the difference between the UV current (I_UV_) and the dark current (I_Dark_) [17].

I_Photo_ = I_UV_ − I_Dark_ (1)

A positive photocurrent was observed under all growth conditions, and its magnitude increased with growth temperature, with the 145 °C device exhibiting the largest photocurrent. This result suggests that increasing the growth temperature improved the generation, transport, and/or collection efficiency of photogenerated carriers. Thus, the ALD growth temperature is an important process parameter affecting not only the intrinsic conductivity of the ZnO film but also its photoinduced carrier-transport characteristics. Figure 2e shows the time-dependent current response measured during 1.0 s UV illumination and for 120 s after the UV light was turned off to reliably evaluate both the fast and slow components of the PPC decay. The current increased rapidly when the UV light was turned on and gradually decreased after the illumination was removed. The 90 °C device returned toward its initial state relatively rapidly, whereas the current decay became slower with increasing growth temperature. In particular, the devices grown at 145 °C retained relatively high current levels at the end of the measurement. This residual current after UV illumination is attributed to PPC in ZnO. Under dark conditions, oxygen molecules adsorbed on the ZnO surface capture electrons and form negatively charged oxygen species. Under UV illumination, photogenerated holes can migrate to the surface and neutralize the adsorbed oxygen species, promoting oxygen desorption and increasing the concentration of free electrons in the ZnO film. After the UV light is turned off, oxygen readsorption and electron recapture proceed relatively slowly; therefore, the current does not immediately return to its initial level [12,13]. In addition to the surface oxygen adsorption–desorption process, carrier trapping and detrapping through defect states in the ZnO film can contribute to the PPC decay [12,14]. Charge associated with different trap states may be released at different rates, producing fast and slow decay components [28]. Additional XPS analysis of the representative ZnO film grown at 90 °C indicated the presence of Zn^2+^–O bonding and oxygen- and hydroxyl-related components associated with Zn–OH, oxygen-vacancy-related states, interstitial oxygen, and hydroxyl species (Figure S1). Because XPS data were available only for the 90 °C sample, these measurements were used only to identify the types of defect-related chemical components present in the ZnO film, rather than to evaluate their growth-temperature dependence. A systematic temperature-dependent XPS investigation will be conducted in future work to further clarify the relationship between defect evolution and PPC behavior. To quantitatively compare the photocurrent decay, the full post-illumination current response recorded for 120 s was fitted using the following double-exponential function [29]:

I(t) = A_1_ exp(−t/τ_1_) + A_2_ exp(−t/τ_2_) (2)

where A_1_ and A_2_ represent the amplitudes of the fast and slow decay components, respectively, and τ_1_ and τ_2_ are their corresponding time constants. The faster component, τ_1_, may involve relatively rapid surface-related processes and/or carrier release from more readily accessible trap states, whereas the slow component, τ_2_, may involve slower oxygen readsorption and/or long-lived carrier trapping processes. These assignments are consistent with commonly reported interpretations of fast and slow relaxation components in ZnO [12,13,14]; however, τ_1_ and τ_2_ are treated here as effective relaxation time constants rather than being uniquely assigned to shallow and deep trap levels. Previous studies have shown that fluctuations in charge trapping/detrapping and interfacial defect states can contribute to low-frequency noise (LFN), which can affect the stability and distinguishability of multilevel conductance states in synaptic and memory devices [30,31,32]. In ZnO, fluctuations in trap occupancy and dynamic surface-oxygen adsorption/desorption may similarly modulate the carrier concentration and potentially influence synaptic-weight stability. Although LFN was not directly measured in the present study, these previous findings provide an additional perspective on the possible relationship between defect-related carrier dynamics and synaptic-state stability. As shown in Figure 2f, τ_1_ was approximately 0.6 s for the 90 °C device, increased to approximately 1.6 s for the 110 and 130 °C devices, and then decreased to approximately 0.8 s for the 145 °C device. This nonmonotonic behavior suggests that the fast decay component is influenced by multiple carrier-relaxation processes rather than by a single trap level. In particular, the 145 °C film exhibited increased surface roughness, larger surface features, and reduced defect-related visible PL intensity. Although these structural and optical changes occur concurrently with the variation in τ_1_, the present AFM and PL measurements do not provide direct evidence that they are responsible for the faster relaxation. Therefore, the reduced τ_1_ at 145 °C is interpreted only as a change in the effective fast relaxation dynamics rather than being directly attributed to improved film connectivity or shallow-trap transport. In contrast, τ_2_ continuously increased with growth temperature and reached a maximum value of approximately 31 s at 145 °C. This result indicates that the slow relaxation process became increasingly dominant in the high-temperature-grown ZnO film, allowing the photogenerated conductivity to persist for a longer period. The simultaneous decrease in τ_1_ and increase in τ_2_ at 145 °C indicates the coexistence of fast and long-lived relaxation processes with different characteristic time scales. However, the present room-temperature transient measurements alone cannot determine whether these components originate from particular shallow, deep, nonradiative, or metastable trap states. The apparently different growth-temperature dependences of the PL intensity and PPC decay may therefore arise because radiative recombination and long-lived carrier trapping do not necessarily involve the same relaxation pathways or defect-related states. The stronger defect-related visible PL observed in the lower-temperature-grown films reflects a greater contribution from radiative sub-bandgap recombination, whereas the prolonged τ_2_ and enhanced PPC observed in the high-temperature-grown films indicate slower carrier-relaxation dynamics. These observations do not, however, permit a unique assignment to specific defect species or trap depths. Therefore, the decrease in PL intensity and the increase in PPC retention with growth temperature are not contradictory, because PL intensity reflects the probability of radiative recombination, whereas PPC is governed primarily by the lifetime and release kinetics of trapped carriers and surface-related relaxation processes. These growth-temperature-dependent photocurrent and PPC characteristics provide the basis for the synaptic responses discussed in the following section. In addition, the electrical and synaptic characteristics of the flexible ZnO/PI device were compared under flat and bending conditions. As shown in Figure S2, slight increases in the dark current, photocurrent, and EPSC were observed during bending, whereas the PPF index remained nearly unchanged. Overall, the device preserved its main optoelectronic and synaptic characteristics under mechanical deformation.

### 3.3. Growth-Temperature-Dependent Short-Term Synaptic Plasticity and PPF Behavior

Figure 3a presents a schematic comparison between the short-term memory behavior of the Al/ZnO/Al optoelectronic synaptic device under UV stimulation and the visual memory process of the human brain. In this device, UV light serves as the presynaptic stimulus, while the EPSC generated by UV exposure represents the postsynaptic response. Similar to how visual information can be retained temporarily after the stimulus disappears, the photogenerated carriers in ZnO do not immediately recombine or become recaptured when the UV light is turned off but remain in the film for a certain period [12,13,14]. Because of these residual photogenerated carriers, the current gradually returns to its initial level after the light is removed, allowing the device to mimic biological short-term memory behavior. Figure 3b shows the EPSC response measured under two consecutive UV pulses, each with a duration of 1.0 s, at a pulse interval of Δt = 1.0 s as a representative PPF response, followed by current decay recorded after the UV light was turned off. In every device, the second pulse produced a larger EPSC than the first pulse because the second stimulus was applied before the carriers generated by the first pulse had completely recombined, detrapped, or been recaptured. Consequently, the residual carriers from the first pulse and the newly generated carriers from the second pulse jointly contributed to the enhanced current, demonstrating a typical PPF response [12,13]. As the ZnO growth temperature decreased from 145 to 90 °C, both the EPSC magnitude and the retention time of the photoinduced current decreased. In particular, the devices grown at lower temperatures exhibited a smaller increase in current during the second pulse, followed by a faster current decay after the stimulation ended. This behavior is consistent with the PPC results discussed in Figure 2e, suggesting that the ability to retain photogenerated carriers became weaker at lower growth temperatures. In contrast, the devices grown at higher temperatures showed larger and longer-lasting EPSC responses, probably because the improved carrier-transport pathways increased the photocurrent, while relatively stable trap states prolonged carrier retention. Because the absolute EPSC magnitude is also influenced by the growth-temperature-dependent dark current and photocurrent, however, an increase in EPSC alone does not necessarily indicate an intrinsic enhancement of synaptic plasticity. Figure 3c compares the first EPSC peak, A_1_, and the second EPSC peak, A_2_, measured at Δt = 1.0 s for each growth temperature. Both A_1_ and A_2_ increased as the growth temperature rose from 90 to 145 °C, with a particularly large increase at 145 °C. A_1_ increased from approximately 80 to 3100 nA, whereas A_2_ increased from approximately 110 to 5900 nA. The difference between A_2_ and A_1_ also increased with growth temperature, indicating that the facilitation induced by the second optical pulse became more pronounced in the devices grown at higher temperatures. This increase in the absolute EPSC difference should be considered together with the simultaneous increase in the overall photocurrent level. The sharp increase in both values between 130 and 145 °C may be related to enhanced photocarrier generation and transport [24], together with prolonged carrier trapping and retention, at the higher growth temperature [12,13,14]. Therefore, Figure 3c demonstrates that increasing the growth temperature enhanced not only the single-pulse EPSC amplitude but also the additional current induced by repeated stimulation. The PPF index was calculated using Equation (3) [33,34]:

PPF = (A_2_/A_1_) × 100 (%) (3)

where A_1_ and A_2_ are the maximum EPSC values induced by the first and second UV pulses, respectively. Unlike the absolute EPSC amplitudes, the PPF index represents a normalized ratio of the second to the first response and therefore substantially reduces the influence of differences in the absolute current level among the devices. Thus, comparison of the PPF index provides a more appropriate measure of short-term synaptic facilitation among devices with different dark-current and photocurrent levels. Figure 3d shows the change in PPF index as the pulse interval, Δt, was varied from 0.1 to 32 s. For each Δt condition, the measurement was performed only after the EPSC had sufficiently returned close to its initial baseline level, thereby minimizing cumulative effects from the preceding pulse sequence and ensuring a comparable initial state for each PPF measurement. For all devices, the PPF value decreased with increasing Δt because the residual carriers generated by the first pulse were gradually recombined, detrapped, or recaptured over time. However, the PPF index decreased more rapidly in the devices grown at lower temperatures, whereas the devices grown at higher temperatures maintained relatively high PPF values even at long pulse intervals. Importantly, this growth-temperature dependence remains evident in the normalized PPF index, indicating that the enhanced facilitation in the higher-temperature-grown devices cannot be explained solely by their larger absolute photocurrent. This trend is consistent with longer carrier-retention and relaxation behavior in the devices grown at higher temperatures. Figure 3e compares the PPF indices measured at Δt = 0.5 and 32 s as a function of the ZnO growth temperature. Under both conditions, the PPF index increased with growth temperature, and the values measured at Δt = 0.5 s were consistently higher than those measured at Δt = 32 s. At Δt = 0.5 s, the PPF index increased from approximately 151% at 90 °C to 204% at 145 °C. At every growth temperature, the PPF index measured at 0.5 s was higher than that measured at 32 s because a large fraction of the carriers generated by the first pulse remained when the interval between the two pulses was shorter. More importantly, the devices grown at higher temperatures still exhibited relatively high PPF indices even at the long interval of 32 s, indicating that the photogenerated carriers remained active for a longer duration and continued to facilitate the response to the subsequent optical stimulus. These results demonstrate that the growth temperature effectively controls the temporal retention and short-term synaptic plasticity of the flexible ZnO optoelectronic synaptic devices.

### 3.4. Optical-Stimulus-Dependent Modulation and Synaptic Learning Characteristics

Figure 4a–d show the EPSC response of Al/ZnO/Al optoelectronic synaptic devices fabricated using ZnO films grown at 90, 110, 130, and 145 °C, respectively, as the UV stimulation time was increased from 0.5 to 3.0 s. For all devices, the EPSC increased with increasing stimulation duration because prolonged UV illumination generated a large number of electron–hole pairs [35,36]. After the UV light was turned off, the current gradually decayed rather than immediately returning to its initial level, indicating the retention of photogenerated carriers through surface-related processes and defect-mediated trapping [14,35,36]. The increase in EPSC and the prolonged post-stimulation current became more pronounced for the devices fabricated at higher growth temperatures. Figure 4e–h show the EPSC responses of the devices grown at 90, 110, 130, and 145 °C, respectively, as the UV light intensity was increased from 66 to 396 μW/cm^2^. Increasing the optical power density produced a progressively larger EPSC by increasing the photocarrier generation rate. The residual current after UV illumination also increased with optical intensity, suggesting that stronger stimulation not only increased the density of mobile carriers but also enhanced carrier occupation of available trap states. The trapped carriers were subsequently released or recaptured over time, resulting in a gradual current decay after the optical stimulus was removed. Figure 4i–l present the EPSC evolution of the devices grown at 90, 110, 130, and 145 °C, respectively, when the number of consecutive UV pulses was increased from 1 to 20. The EPSC increased stepwise during repeated optical stimulation because each successive pulse generated additional carriers before the carriers produced by the preceding pulses had completely recombined, detrapped, or been recaptured. This cumulative response represents an optical learning process in which repeated stimulation progressively strengthens the synaptic weight. 

After completion of the pulse sequence, devices subjected to a larger number of pulses exhibited higher residual currents and slower relaxation, indicating enhanced carrier accumulation and retention. A comparison among the four growth temperatures shows that the effects of stimulation duration, light intensity, and pulse number were consistently stronger in the devices fabricated with ZnO films grown at higher temperatures. Under comparable stimulation conditions, the 90 °C device exhibited the smallest EPSC and the fastest current relaxation, whereas the 145 °C device showed the largest EPSC and the most persistent post-stimulation current. The enhanced responses at higher growth temperatures may result from the combined effects of improved carrier-transport pathways [24] and an increased contribution from long-lived trapping or surface-relaxation processes [12,13]. This interpretation is consistent with the growth-temperature-dependent photocurrent and persistent photoconductivity characteristics discussed in Figure 2. Overall, Figure 4 demonstrates that the EPSC can be effectively modulated by the duration, intensity, and number of optical stimuli. Stronger stimulation and higher ZnO growth temperatures increase the EPSC and prolong its retention, enabling tunable synaptic plasticity and memory behavior.

### 3.5. Growth-Temperature-Dependent Learning, Forgetting and Visual Memory Characteristics

Figure 5a compares the learning and forgetting characteristics of Al/ZnO/Al optoelectronic synaptic devices fabricated with ZnO films grown at different temperatures. During the first learning process, 100 UV pulses were applied with a pulse width of 0.1 s and a duty cycle of 50%, after which the natural decay of EPSC was recorded as the first forgetting process. To compare the memory characteristics of the devices independently of their different absolute current levels, 70% of the maximum EPSC obtained during the first learning process was defined as the learning and forgetting threshold. Thus, the threshold represents a normalized retention criterion rather than a fixed absolute current value. As the ZnO growth temperature increased, the maximum EPSC induced by repeated UV stimulation increased, and the current persisted for a longer period after the stimulation was terminated. The increase in the maximum EPSC partly reflects the higher photocurrent of the devices grown at higher temperatures and therefore, by itself, does not necessarily represent improved intrinsic memory characteristics. In contrast, the time required for the EPSC to decrease to 70% of its own maximum value provides a relative measure of memory retention that minimizes the influence of differences in absolute current magnitude. The device grown at 90 °C reached the forgetting threshold within approximately 0.1 s, whereas the device grown at 145 °C maintained an EPSC above the threshold for more than approximately 65 s. The substantially longer retention time at the same normalized threshold indicates that the slower forgetting in the higher-temperature-grown devices cannot be attributed solely to their larger initial current. This growth-temperature-dependent behavior indicates that the devices fabricated at higher temperatures exhibited enhanced photocarrier retention and slower memory decay. During the second learning process, all devices reached the same relative threshold with fewer pulses than during the first learning process. Thus, prior optical stimulation reduced the number of pulses required for relearning, mimicking the learning-experience behavior of biological synapses. A slower current decay was also observed during the second forgetting process, and this effect became more pronounced at higher temperatures. Residual carriers remaining after the first learning cycle and additional occupation of relatively stable trap states during the second stimulation may facilitate faster relearning and longer memory retention. Among the devices, the 145 °C device exhibited the largest EPSC and the slowest forgetting behavior, thereby showing the most pronounced long-term memory characteristics. Importantly, the latter conclusion is based on the normalized retention behavior rather than solely on the absolute EPSC magnitude. Figure 5b presents the parameters extracted by fitting the learning and forgetting curves using the Wickelgren function [36,37]:

I(t) = λ × (1 + βt)^−ψ^ (4)

where (I(t)) represents the remaining memory response at time t, λ denotes the initial learning level, β is related to the temporal decay rate, and ψ describes the degree of forgetting. The Wickelgren function was employed here as an empirical model to quantitatively compare the learning–forgetting and memory-retention behavior among the devices, rather than to represent a specific microscopic carrier-trapping mechanism. To further minimize the influence of the different absolute EPSC levels among the devices, the forgetting curves were normalized to the maximum EPSC of each learning cycle before comparison of the decay behavior. The fitting results show that the normalized memory response decayed more slowly with increasing ZnO growth temperature, accompanied by a decrease in ψ. In addition, the second learning–forgetting cycle exhibited slower normalized memory decay than the first cycle, particularly for the higher-temperature-grown devices. These results quantitatively indicate that repeated UV stimulation facilitated relearning and reduced the subsequent memory-decay rate, even after differences in the absolute current level were removed by normalization. To visualize these growth-temperature-dependent learning and forgetting characteristics at the array level, Figure 5c illustrates the optical learning and forgetting procedure using a robot-shaped mask composed of a 5 × 5 pixel pattern. For this experiment, 25 physically distinct and adjacent devices were selected from the same wafer and arranged as a 5 × 5 pixel configuration. The robot-shaped UV pattern was applied to the corresponding devices, and 100 UV pulses were applied to each selected pixel device. The current response of each device was then individually measured. After the UV illumination was turned off, the gradual disappearance of the stored image was monitored as a function of time. Figure 5d presents the first and second learning–forgetting process reconstructed from the individually measured responses of the 25 adjacent devices forming the 5 × 5 pixel configuration for ZnO films grown at 90, 110, 130, and 145 °C. The state of each pixel was evaluated using 70% of the maximum EPSC as the memory threshold, and a pixel was regarded as learned when its current remained above this value. As the growth temperature increased, the robot pattern remained distinguishable for a longer period during the forgetting process. This behavior was evident for the 145 °C device, in which the stored image retained relatively high contrast and faded more slowly than those in the other devices. In contrast, the pattern stored in the 90 °C device disappeared rapidly after the UV illumination was removed. To quantitatively support the visual-memory behavior, the average normalized retention of the stimulated pixels was evaluated as a function of retention time for each growth temperature. As shown in Figure S3, the normalized pixel retention decreased with time for all devices, while the higher-temperature-grown devices, particularly the 145 °C device, retained a larger fraction of the initially learned response. The second learning cycle also showed improved retention compared with the first cycle, quantitatively supporting the relearning behavior observed in the 5 × 5 visual-memory maps. The visual-memory results are consistent with the growth-temperature-dependent EPSC, PPC, and learning–forgetting characteristics described above. Devices grown at higher temperatures retained photogenerated carriers for longer periods, resulting in slower image degradation, whereas faster carrier relaxation in the lower-temperature devices caused the stored pattern to disappear more rapidly. These differences in retention are consistent with the growth-temperature-dependent changes in photocurrent relaxation and PPC observed in Figure 2, rather than being attributed directly to the increase in AFM roughness or surface-feature size. The accompanying changes in defect-related visible PL suggest that the radiative recombination environment also varies with growth temperature; however, the specific defect states responsible for the long-lived PPC cannot be uniquely identified from the present measurements. At all growth temperatures, the second learning process reproduced the robot pattern more rapidly than the first learning process, and the image remained visible for a longer period during the second forgetting process. This behavior demonstrates that the prior learning history was retained by the device and facilitated subsequent relearning of the same optical pattern. Overall, Figure 5 establishes a direct relationship between the quantitatively evaluated learning-forgetting behavior of individual devices and the visual-memory characteristics of the corresponding 5 × 5 pixel configurations. By controlling the ALD growth temperature of ZnO, the memory-retention time can be systematically adjusted; devices grown at high temperatures are advantageous for longer-term visual-memory retention, whereas those grown at lower temperatures may be suitable for rapidly fading short-term memory. These results demonstrate that the ZnO growth temperature is an effective process parameter for controlling optical learning, relearning, forgetting, and image-retention characteristics in flexible optoelectronic synaptic devices.

To further place the present device performance in the context of previously reported ZnO-based optoelectronic synapses, Table 1 compares representative devices in terms of device structure, fabrication method and substrate, optical response, EPSC, PPF, and retention characteristics. The present ALD-grown Al/ZnO/Al device exhibits a PPF index of approximately 204% at Δt = 0.5 s and a retention time of approximately 65 s, while also offering direct low-temperature growth on a flexible PI substrate. Compared with previously reported ZnO thin-film, nanoparticle, microtube, nanowire, and printed devices, these results demonstrate that the present approach provides competitive synaptic performance together with the practical advantage of low-temperature ALD compatibility with flexible substrates.

## 4. Conclusions

In this study, Al/ZnO/Al optoelectronic synaptic devices were fabricated on PI substrates using ZnO films grown by ALD at temperatures from 90 to 145 °C. The effect of the ZnO growth temperature on the structural, optical, electrical, and synaptic characteristics was systematically investigated. As the growth temperature increased, the dark current, photocurrent, and PPC increased, indicating improved carrier transport and enhanced photocarrier retention. The higher-temperature-grown device also exhibited larger EPSC values and slower post-illumination decay. The EPSC was effectively modulated by the UV stimulation duration, intensity, and pulse number. PPF remained higher with longer pulse intervals for devices grown at higher temperatures, demonstrating prolonged retention of photogenerated carriers. Repeated UV stimulation enabled faster relearning and slower forgetting, mimicking learning-experience behavior. The 145 °C device exhibited the largest EPSC and the longest memory retention. Furthermore, 5 × 5 visual memory tests showed that the retention and fading of stored optical patterns could be controlled by the ZnO growth temperature. Overall, growth-temperature engineering of ALD ZnO provides an effective approach for tuning synaptic plasticity and visual-memory retention in flexible optoelectronic devices, with potential applications in wearable neuromorphic systems and artificial visual memory.

## Figures and Tables

**Figure 1:**
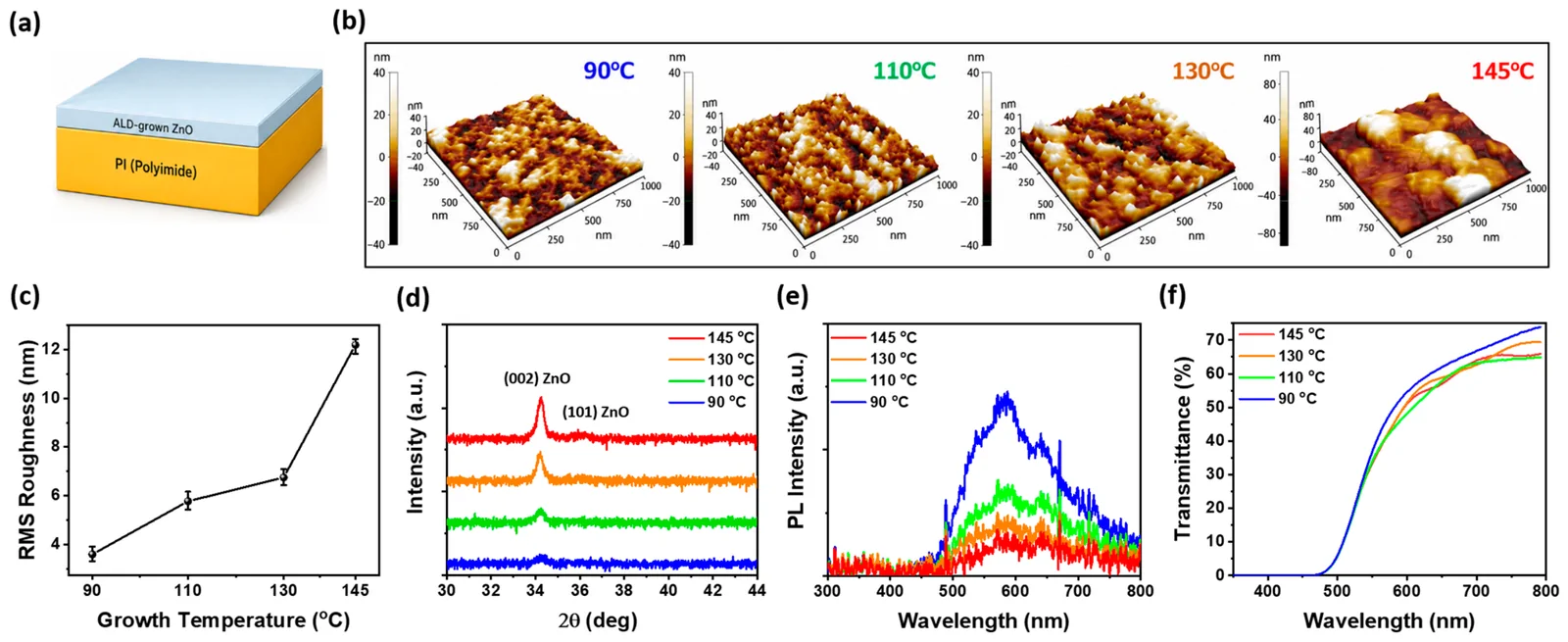
(**a**) Schematic structure of the ZnO/PI sample. (**b**) Three-dimensional AFM images of the ZnO films grown at 90, 110, 130, and 145 °C. (**c**) RMS surface roughness as a function of growth temperature. (**d**) ω–2θ XRD patterns of the ZnO films grown at 90–145 °C. (**e**) Room-temperature PL spectra showing broad defect-related visible emission. (**f**) UV–visible transmittance spectra of the ZnO/PI samples grown at different temperatures.

**Figure 2:**
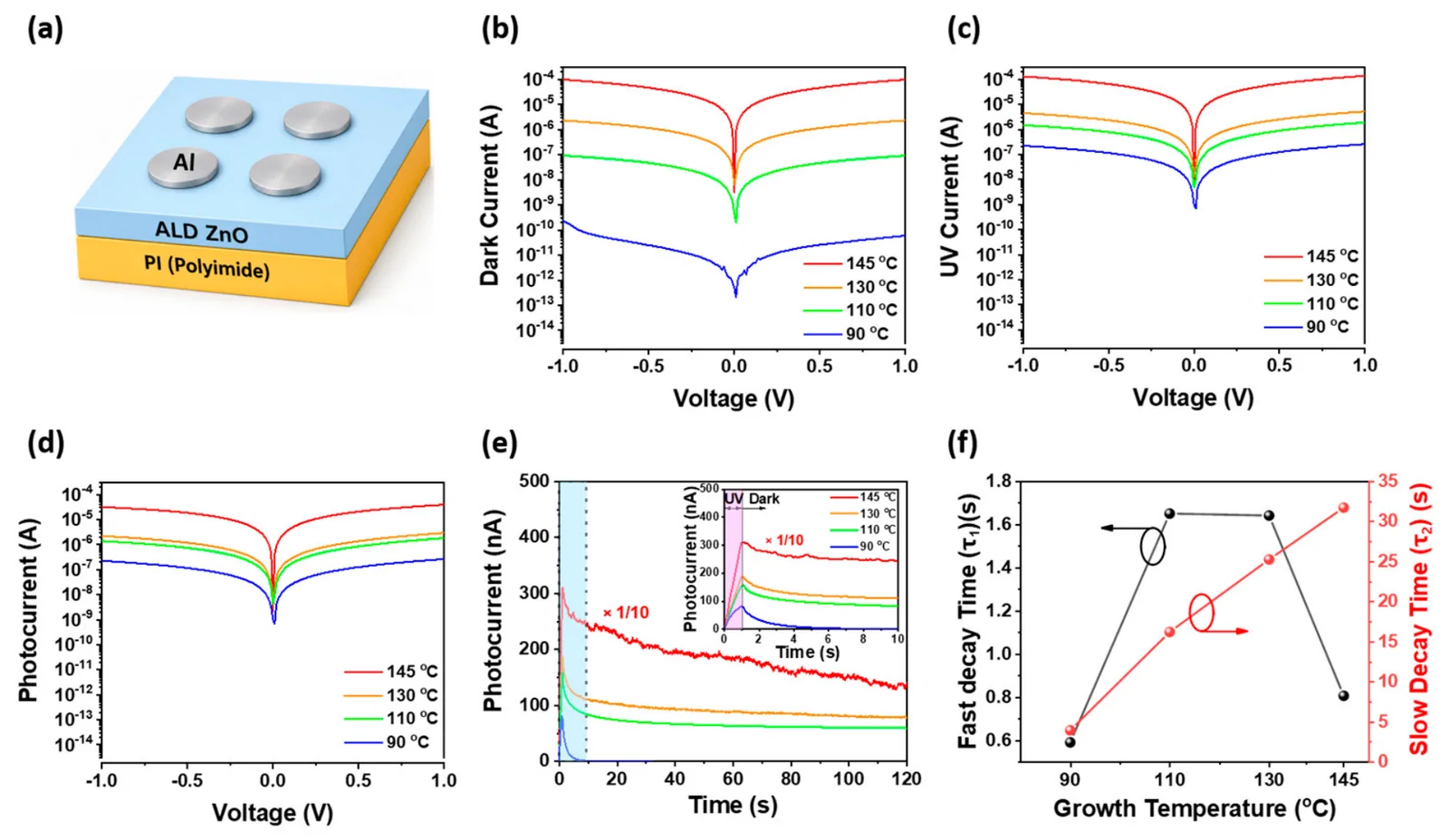
(**a**) Schematic illustration of the planar Al/ZnO/Al device on a PI substrate. (**b**) Dark current–voltage characteristics and (**c**) current–voltage characteristics under 365 nm UV illumination for devices fabricated with ZnO films grown at 90, 110, 130, and 145 °C. (**d**) Photocurrent characteristics calculated from the difference between the UV-illuminated and dark currents. (**e**) Time-dependent current responses measured at an operating voltage of 1.0 V during and after UV illumination over a measurement period of 120 s, showing persistent photoconductivity behavior; the inset shows an enlarged view of the initial 0–10 s region to highlight the rapid photocurrent response during and immediately after UV illumination. (**f**) Extracted fast and slow decay time constants, τ_1_ and τ_2_, obtained by fitting the post-illumination current decay with a double-exponential function.

**Figure 3:**
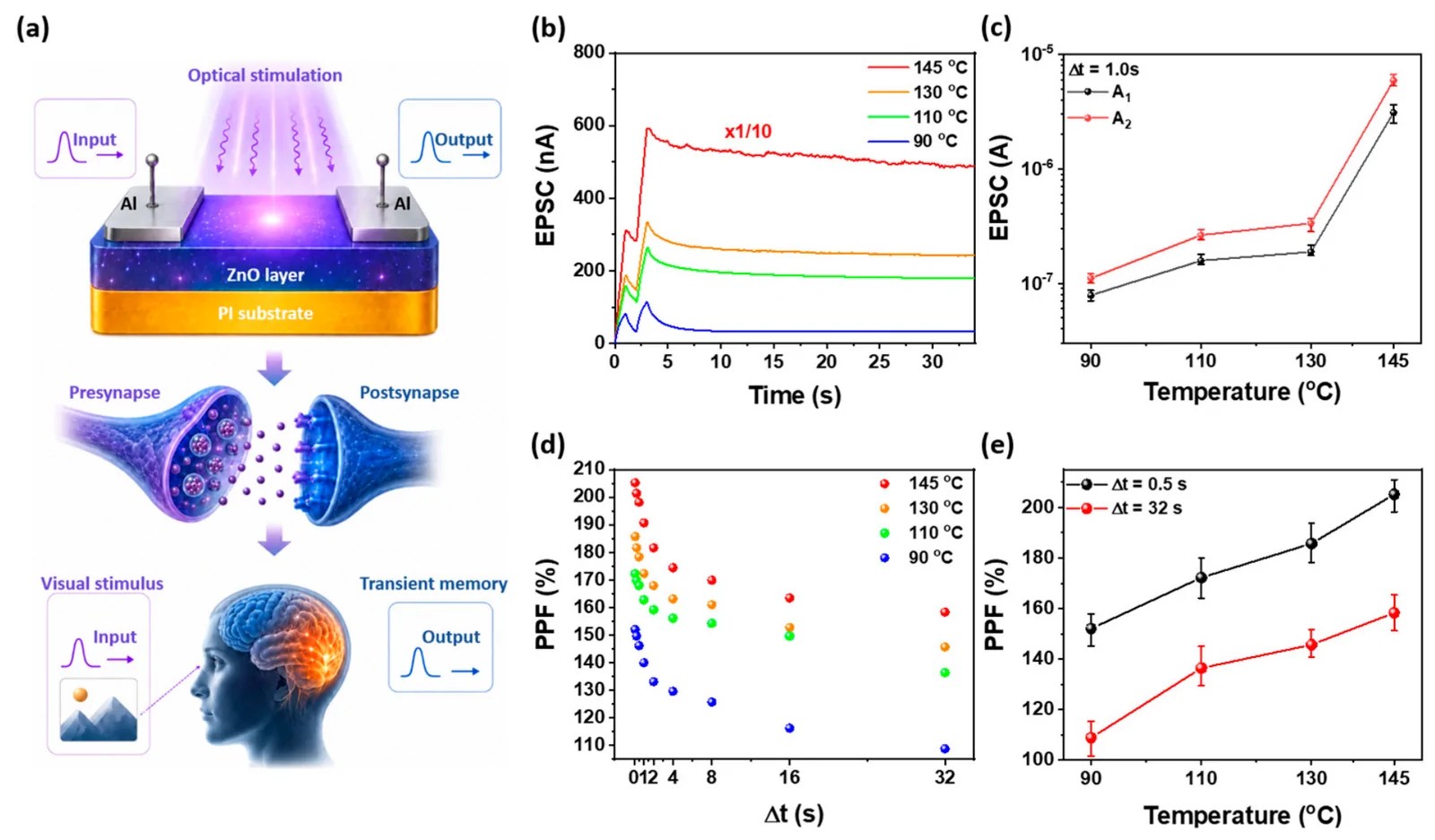
(**a**) Schematic illustration comparing the UV-induced synaptic response of the Al/ZnO/Al device with short-term visual memory in the human nervous system. (**b**) EPSC responses induced by two consecutive 365 nm UV pulses with a pulse duration of 1.0 s, measured at an operating voltage of 1.0 V. (**c**) First and second EPSC peak amplitudes, A_1_ and A_2_, measured at a pulse interval of Δt = 1.0 s as a function of ZnO growth temperature. (**d**) PPF index as a function of pulse interval for devices fabricated with ZnO films grown at 90, 110, 130, and 145 °C, measured at an operating voltage of 1.0 V. (**e**) Comparison of the PPF indices measured at Δt = 0.5 and 32 s as a function of ZnO growth temperature.

**Figure 4:**
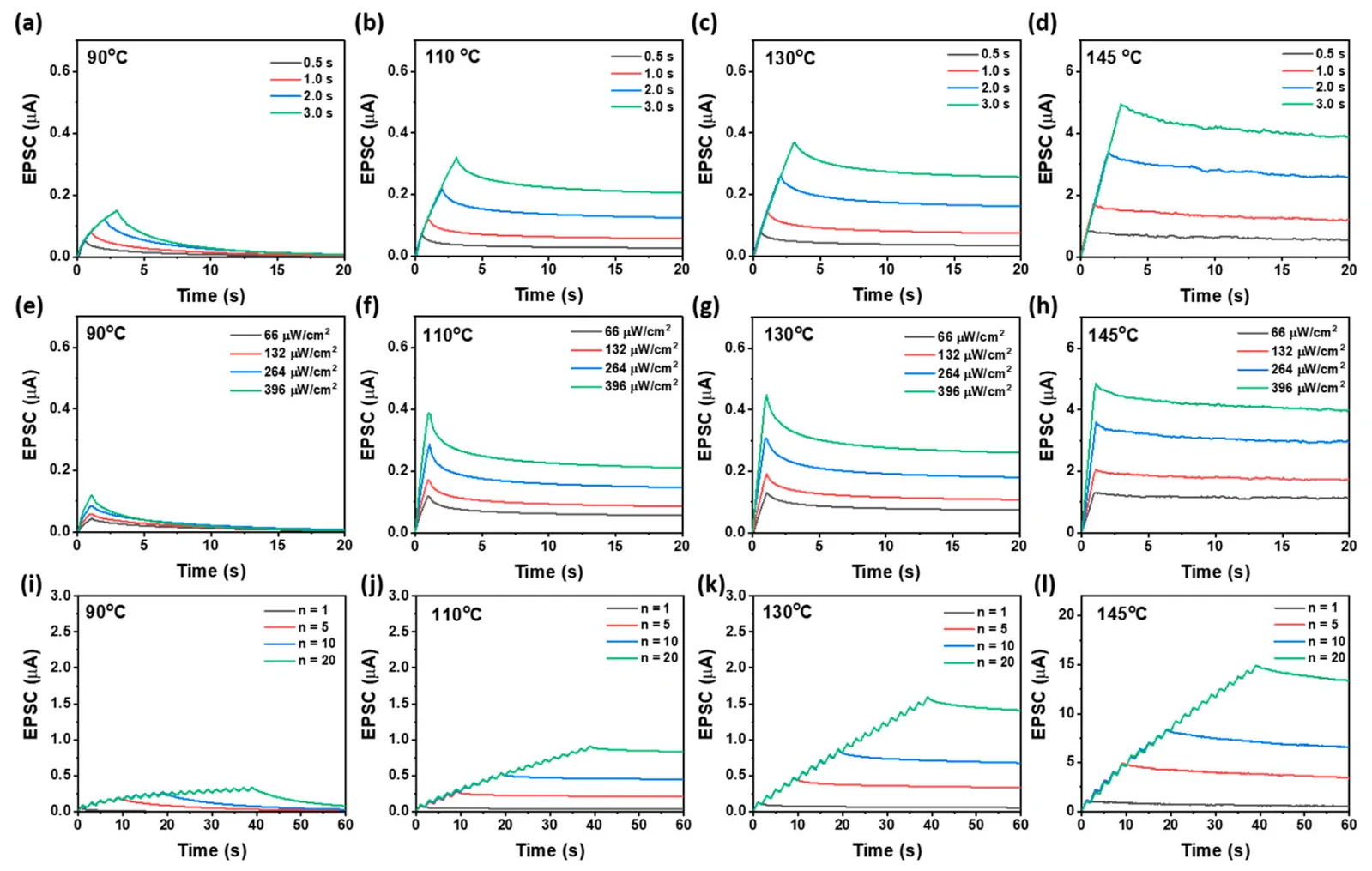
EPSC responses of flexible Al/ZnO/Al optoelectronic synaptic devices fabricated with ZnO films grown at 90, 110, 130, and 145 °C, measured at an operating voltage of 1.0 V. (**a**–**d**) Dependence on UV stimulation duration (0.5–3.0 s), (**e**–**h**) UV light intensity (66–396 μW/cm^2^), and (**i**–**l**) number of UV pulses (1–20).

**Figure 5:**
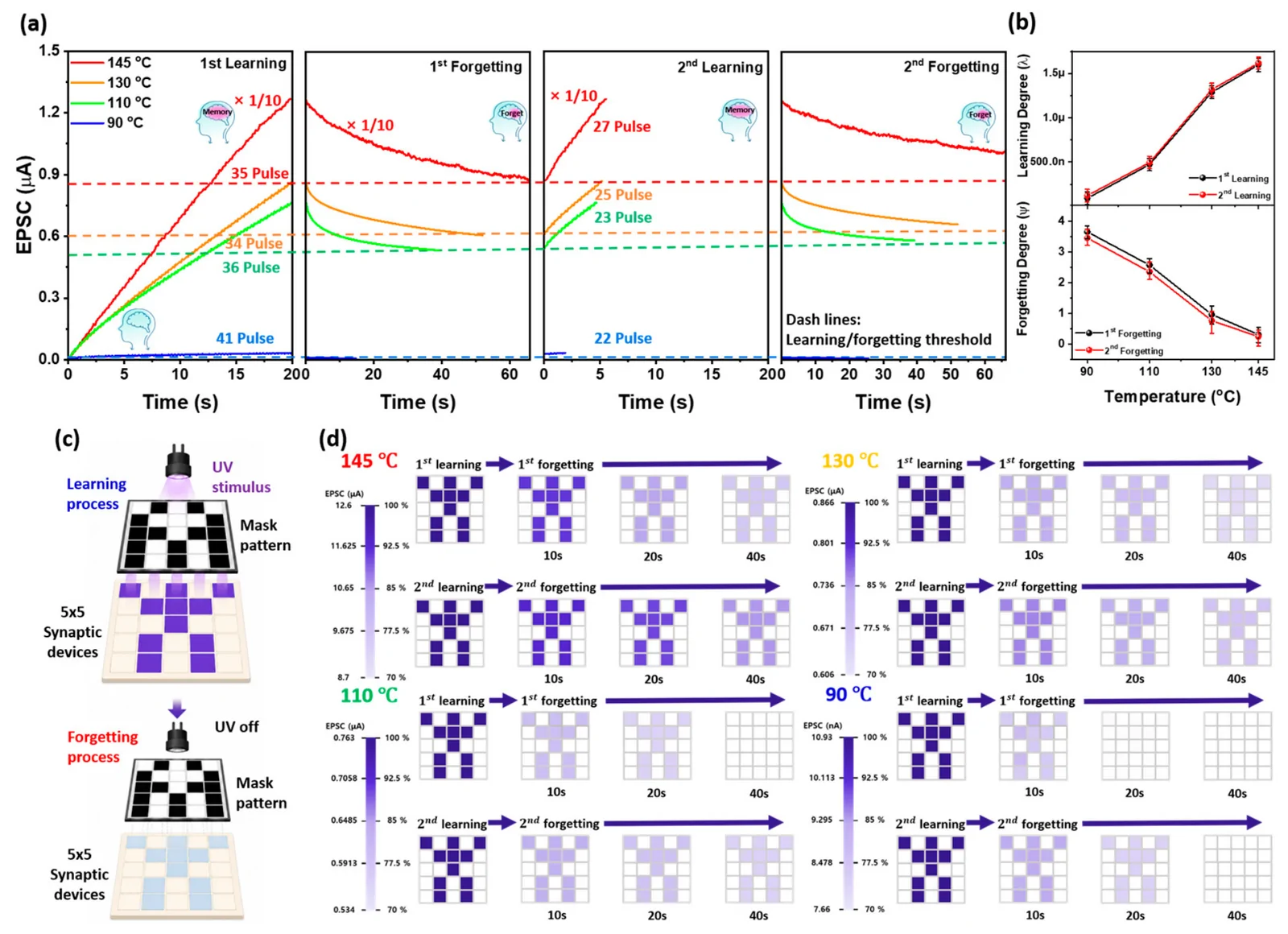
(**a**) First and second learning–forgetting processes under repeated UV stimulation, measured at an operating voltage of 1.0 V. (**b**) Extracted Wickelgren fitting parameters for the learning and forgetting curves. (**c**) Schematic of the optical learning and forgetting process using a robot-shaped 5 × 5 mask. (**d**) Growth-temperature-dependent retention and fading of the learned robot pattern during the first and second learning–forgetting cycles measured at an operating voltage of 1.0 V.

**Table 1:** Performance comparison of previously reported ZnO-based optoelectronic synaptic devices.

Device Structure	Fabrication /Substrate	Optical Response	EPSC	PPF	Retention	Ref.
ZnO nanoparticles	Spin coating /Sapphire	UV	μA-level	180% at Δt = 0.5 s	>350 s	[3]
ZnO nanowires	RF sputtering /Flexible PDMS	UV	10 μA	NR	STM-LTM ~120 s	[13]
ZnO thin film	RF sputtering /Rigid substrate	UV I_on_/I_off_ > 10^3^		NR	STM-LTM	[38]
A-ZnO microtube	Microtube /Graphene	UV	~5 nA	180%	33.8 s	[39]
Pt/ZnO/Pt	ZnO NPs /Rigid substrate	UV	800 nS	NR	2500 s	[40]
Inkjet-printed ZnO	Inkjet printing /Transparent substrate	UV		>220%	146 s	[41]
Al/ZnO/Al	ALD at 90–145 °C /Flexible PI	365 nm UV	~1.0 μA	204% at Δt = 0.5 s	65 s	This work

## Data Availability

The data presented in this study are available on request from the corresponding author. The data are not publicly available due to privacy concerns.

## Supplementary Materials

The following supporting information can be downloaded at: https://www.mdpi.com/article/10.3390/s26196022/s1, Figure S1: XPS Analysis of the ZnO Film; Figure S2: Electrical and optoelectronic characteristics of the flexible Al/ZnO/Al device; Figure S3: Quantitative visual-memory retention of the 5 × 5 pixel configurations.
