# Supplementary material for: Growth-Temperature-Dependent Synaptic Plasticity in ALD-ZnO Optoelectronic Synaptic Devices on Polyimide Substrates
Source: Sensors (Basel). 2026 Sep 23;26(19):6022. doi: 10.3390/s26196022 (PMC13667250; doi:10.3390/s26196022)
Supplement: Supplementary file 1 [file sensors-26-06022-s001.zip › sensors-4525991-supplementary.pdf]

Growth-Temperature-Dependent Synaptic Plasticity in ALD-ZnO Optoelectronic Synaptic Devices on Polyimide Substrates

JunHyeong Park[1], JungBeen Cho[1], SeungMin Song[1], Seyeon Tak[1], Yoon Seok Kim[2], Sung-Nam Lee[1,2*]

[1]Department of Department of Nano and Semiconductor Engineering, Tech University of Korea, Siheung 15073, Republic of Korea

[2]Department of Semiconductor Engineering, Tech University of Korea, Siheung 15073, Republic of Korea

Correspondence: snlee@tukorea.ac.kr; Tel.: +82-31-8041-0721

S1. XPS Analysis of the ZnO Film

S2. Evaluation of Electrical, Photoresponsive, and Synaptic Stability of the Flexible Al/ZnO/Al Device under Bending Conditions

S3. Quantitative analysis of visual-memory retention in the 5 × 5 ZnO/PI synaptic pixel configuration.

S1. XPS Analysis of the ZnO Film

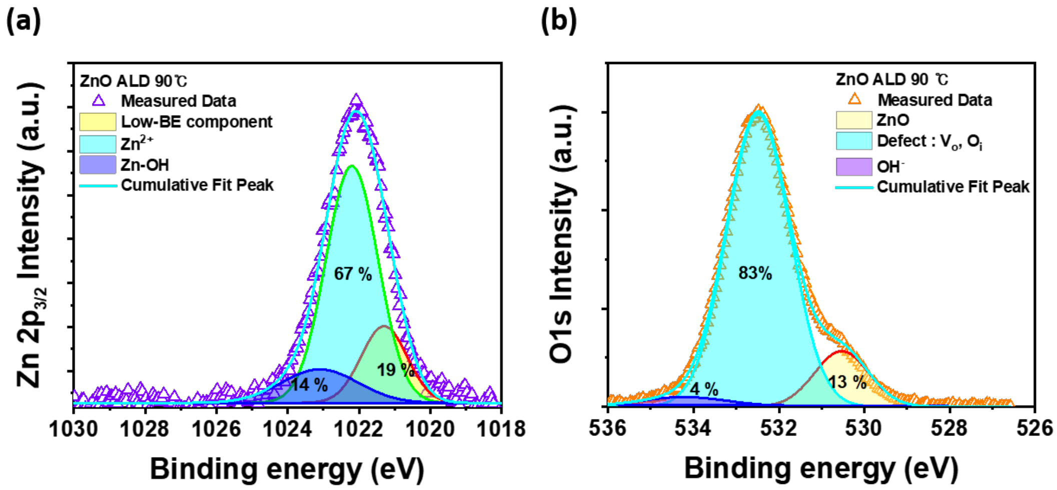

Figure S1. XPS spectra of the ZnO film grown at 90 °C: (a) Zn 2p core-level spectrum and (b) O 1s core-level spectrum, showing Zn^{2+}–O bonding and oxygen- and hydroxyl-related chemical components associated with Zn–OH, oxygen-vacancy-related states, interstitial oxygen, and hydroxyl species.

The chemical bonding states of the representative ZnO film grown at 90 °C were examined by X-ray photoelectron spectroscopy (XPS). The Zn 2p spectrum confirms the characteristic Zn^{2+} chemical state of ZnO, while the O 1s spectrum contains multiple components associated with lattice oxygen and oxygen- and hydroxyl-related species. The deconvoluted O 1s peaks indicate the presence of defect-related chemical components, including oxygen-vacancy-related states, interstitial oxygen, Zn–OH, and hydroxyl species. These results provide supporting evidence for the presence of oxygen-related and surface-related defect states that may participate in carrier trapping and surface reactions relevant to persistent photoconductivity. Because XPS was measured only for the 90 °C sample, the results are used solely to identify the types of chemical and defect-related components present in the ZnO film and are not interpreted as evidence of their growth-temperature dependence.

S2. Evaluation of Electrical, Photoresponsive, and Synaptic Stability of the Flexible Al/ZnO/Al Device under Bending Conditions

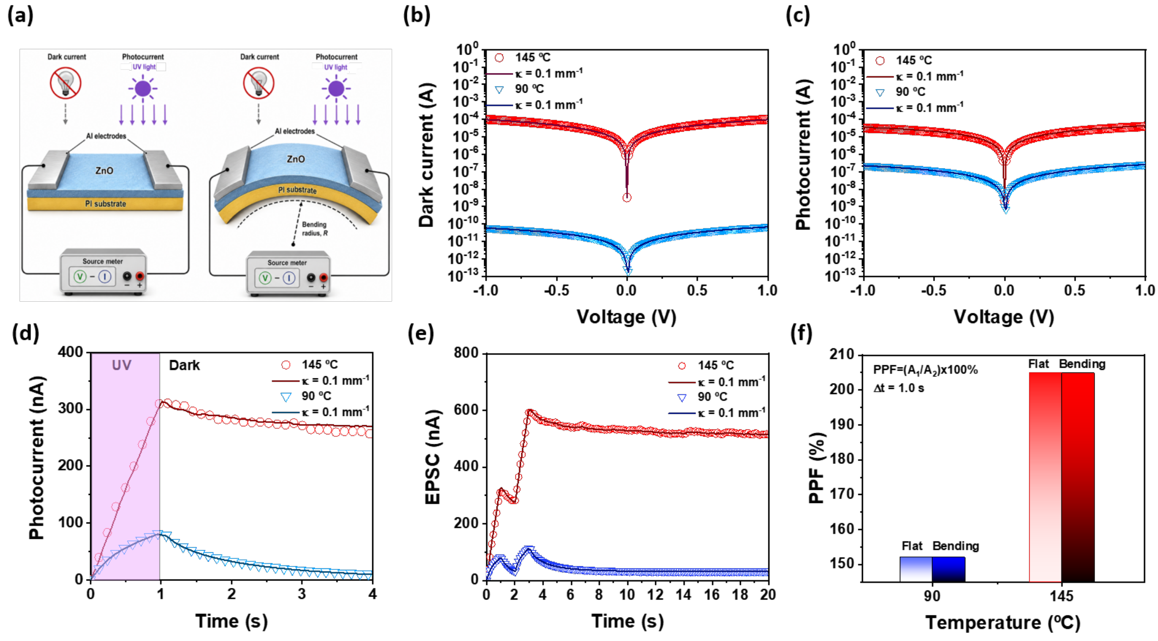

Figure S2. Electrical and optoelectronic characteristics of the flexible Al/ZnO/Al device measured under flat and bending conditions measured at an operating voltage of 1.0 V: (a) measurement configuration, (b) dark-current characteristics, (c) photocurrent characteristics, (d) time-dependent photoresponse; (e) EPSC responses induced by two consecutive optical stimuli, and (f) PPF indices measured at $\Delta t = 1.0$ s under flat and bending conditions.

To evaluate the mechanical flexibility of the device, its electrical and optoelectronic characteristics were compared under flat and bending conditions. As shown in Figure S2, the overall dark-current and photocurrent behaviors were maintained during bending, although both the dark current and photocurrent exhibited a slight increase in the bent state compared with the flat state. Here, κ represents the bending curvature ($\kappa=1/R$, R: radius of curvature) degree of bending applied to the flexible ZnO/PI device, with a larger κ corresponding to a greater bending deformation. The time-dependent photoresponse also remained similar under mechanical deformation, while the photocurrent decay after UV illumination became slightly slower under bending, indicating a modest increase in photocarrier retention. In contrast, the PPF index measured at $\Delta t = 1.0$ s showed almost no change between the flat and bending conditions, demonstrating that the short-term synaptic facilitation behavior was well preserved during mechanical deformation. These minor variations may originate from strain-induced changes in the ZnO channel, electrode contact, or interfacial configuration. Nevertheless, the preservation of the main electrical, photoresponsive, and synaptic characteristics confirms that the flexible Al/ZnO/Al device remained operational under bending conditions.

S3. Quantitative analysis of visual-memory retention in the 5 × 5 ZnO/PI synaptic pixel configuration.

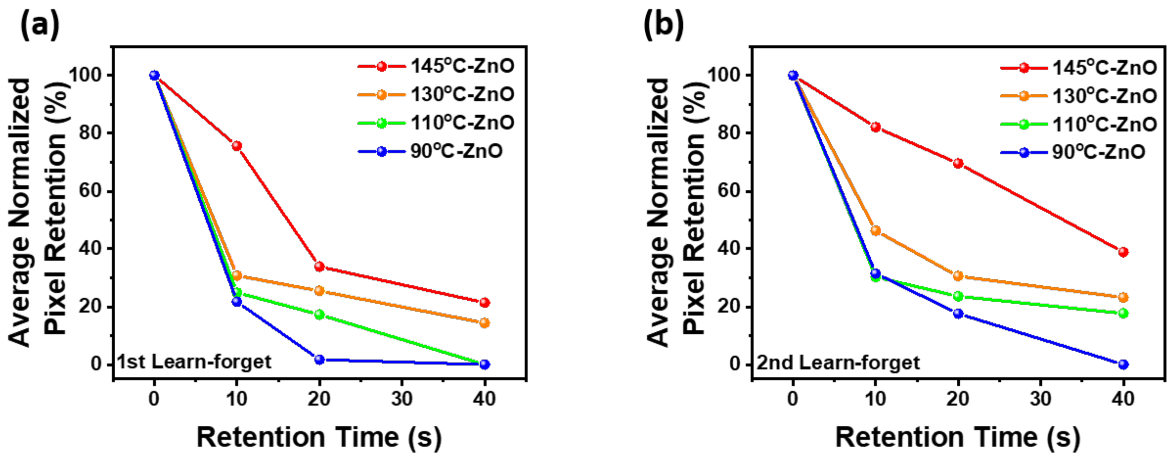

Figure S3. Quantitative visual-memory retention of the 5 × 5 pixel configurations after the (a) first and (b) second learning cycles. The average normalized retention of the stimulated pixels is plotted as a function of retention time for ZnO devices grown at 90, 110, 130, and 145 °C.

Figure S3 was derived from the pixel-resolved data presented in Figure 5(d). For each growth temperature, the EPSC value of every stimulated pixel in the 5 × 5 configuration was normalized to its corresponding maximum post-learning EPSC. The normalized retention of each pixel at 10, 20, and 40 s was then calculated, and the average value of the stimulated pixels was used to represent the overall visual-memory retention at each time point. The same procedure was applied separately to the first and second learning–forgetting cycles. As shown in Figure S3, the average normalized retention decreased with time for all devices, whereas higher-temperature-grown devices, particularly the 145 °C device, exhibited slower decay and higher retained responses. The second learning cycle also showed improved retention compared with the first cycle, supporting the enhanced relearning and memory-retention behavior observed in Figure 5d.
